# The Role of AMPARs in the Maturation and Integration of Caudal Ganglionic Eminence-Derived Interneurons into Developing Hippocampal Microcircuits

**DOI:** 10.1038/s41598-019-41920-9

**Published:** 2019-04-01

**Authors:** G. Akgül, D. Abebe, X. Q. Yuan, K. Auville, C. J. McBain

**Affiliations:** Porter Neuroscience Research Centre, Rm3C903, Lincoln Drive, Eunice Kennedy Shriver National Institute of Child Health and Human Development, Bethesda, MD 20892 USA

## Abstract

In the hippocampal CA1, caudal ganglionic eminence (CGE)-derived interneurons are recruited by activation of glutamatergic synapses comprising GluA2-containing calcium-impermeable AMPARs and exert inhibitory regulation of the local microcircuit. However, the role played by AMPARs in maturation of the developing circuit is unknown. We demonstrate that elimination of the GluA2 subunit (GluA2 KO) of AMPARs in CGE-derived interneurons, reduces spontaneous EPSC frequency coupled to a reduction in dendritic glutamatergic synapse density. Removal of GluA1&2&3 subunits (GluA1-3 KO) in CGE-derived interneurons, almost completely eliminated sEPSCs without further reducing synapse density, but increased dendritic branching. Moreover, in GluA1-3 KOs, the number of interneurons invading the hippocampus increased in the early postnatal period but converged with WT numbers later due to increased apoptosis. However, the CCK-containing subgroup increased in number, whereas the VIP-containing subgroup decreased. Both feedforward and feedback inhibitory input onto pyramidal neurons was decreased in GluA1-3 KO. These combined anatomical, synaptic and circuit alterations, were accompanied with a wide range of behavioural abnormalities in GluA1-3 KO mice compared to GluA2 KO and WT. Thus, AMPAR subunits differentially contribute to numerous aspects of the development and maturation of CGE-derived interneurons and hippocampal circuitry that are essential for normal behaviour.

## Introduction

Hippocampal GABAergic local circuit inhibitory interneurons target distinct domains of their postsynaptic targets to gate incoming excitatory input, control firing of principal neurons, and pace synchronized activity among neurons in both neonatal^1^ and adult brain^2–5^. To date more than 20 subgroups of GABAergic interneurons have been identified in the rodent CA1 hippocampus^6,7^. Fate mapping strategies have revealed that distinct interneuron subtypes are derived from either the medial- (MGE) (e.g. parvalbumin- (PV), somatostatin- (SOM), and neuronal nitric oxide synthase- (nNOS) containing interneurons) or caudal-ganglionic eminence (CGE) precursor pools (e.g., cholecystokinin- (CCK), vasoactive intestinal polypeptide (VIP), reelin- (RE) and calretinin- (CR) containing interneurons)^6–10^. On exiting proliferation interneurons migrate tangentially towards the hippocampus using both genetic^11^ and environmental cues^12^. Cell numbers are then consolidated through programmed apoptotic cell death^13–16^ that in part relies on activity dependent regulation through a calcineurin-dependent mechanism^15^.

Within cortical microcircuits fast excitatory synaptic transmission is largely provided by AMPA-, kainate- and NMDA-preferring glutamate receptors. Although glutamate receptor expression occurs during proliferative migratory stages^17^ most excitatory synapses are not fully functional until the early postnatal period^18^, suggesting roles for glutamate receptors in migration, integration and survival of interneurons that are distinct from their canonical synaptic roles. Indeed, ablation or alteration of glutamate receptors in principal neurons either during embryogenesis, or early development, have major consequences at both anatomical and physiological levels that ultimately impact emerging nascent cortical microcircuits^19–23^. In inhibitory interneurons elimination of glutamate receptors either during embryogenesis or postnatally has mixed impact on cellular and synaptic features. Elimination of NMDAR subunits alters AMPAR synapse development and circuit integration of both hippocampal and neocortical interneurons^10,24^. In Neurogliaform cells (NGFC) of the hippocampal stratum lacunosum-moleculare (SLM) elimination of NMDARs causes cell hypertrophy and alters both pre- and postsynaptic properties of remaining AMPAR-mediated synaptic transmission^10^. Furthermore, postnatal ablation of NMDARs from primarily PV-containing interneurons has been suggested to confer “schizophrenic-like” properties to the maturing circuit^25^. These data suggest cell-type specific, complex roles for glutamate receptors in interneuron development^10^.

In this study, we investigated the anatomical, physiological and behavioural consequences resulting from loss of GluA subunits in CGE-derived interneurons during development. To understand the importance of AMPAR-mediated recruitment of CGE-derived interneurons in local microcircuits, we generated a conditional knockout line in which AMPARs were eliminated (knockout of GluA1&2&3 subunits). Furthermore, to elucidate potential regulatory roles of GluA2 subunits particularly in synaptic maturation, we generated another conditional knockout line in which AMPARs were converted from GluA2-containing Ca^2+^-impermeable AMPARs to GluA2-lacking Ca^2+^-permeable AMPARS (by GluA2 elimination). We found that loss of GluA2 alone reduced sEPSCs onto CGE-derived interneurons, coupled to a reduction in the density of anatomically identified glutamatergic synapses. Elimination of GluA1-3 resulted in near complete loss of sEPSCs, concomitant with cell hypertrophy. Furthermore, elimination of GluA1-3 differentially impacted interneuron survival; increasing the number of CCK-containing cells but decreasing the number of VIP-containing interneurons. The consequent decreased excitatory drive onto CGE-derived interneurons eroded both feedforward and feedback inhibition onto CA1 pyramidal neurons, resulting in an anxiety-like phenotype and deficits in social interaction and Morris water maze performance.

## Results

### Loss of GluA2 and GluA1-3 decreases the frequency of glutamatergic synaptic input onto CGE-derived interneurons

Glutamate receptor containing synapses onto CA1 hippocampal CGE-derived inhibitory interneurons typically comprise GluA2-containing Ca^2+^-impermeable AMPA receptors (GluA) and GluN2B-containing NMDARs^26^. Although the synaptic properties of these glutamate receptors have been studied in detail^7,18,26^, little is known about the role(s) played by glutamate receptors on CGE-derived interneurons during development. To investigate the role of AMPAR subunits in the development of nascent CGE-derived inhibitory interneuron synapses and circuits we generated two CGE-derived interneuron specific AMPAR knockout (KO) lines: a GluA2 KO that converted their AMPARs into GluA2-lacking, Ca^2+^-permeable AMPARs, and a GluA1-3 triple KO that eliminated almost the entire AMPAR pool.

**Figure 1:**
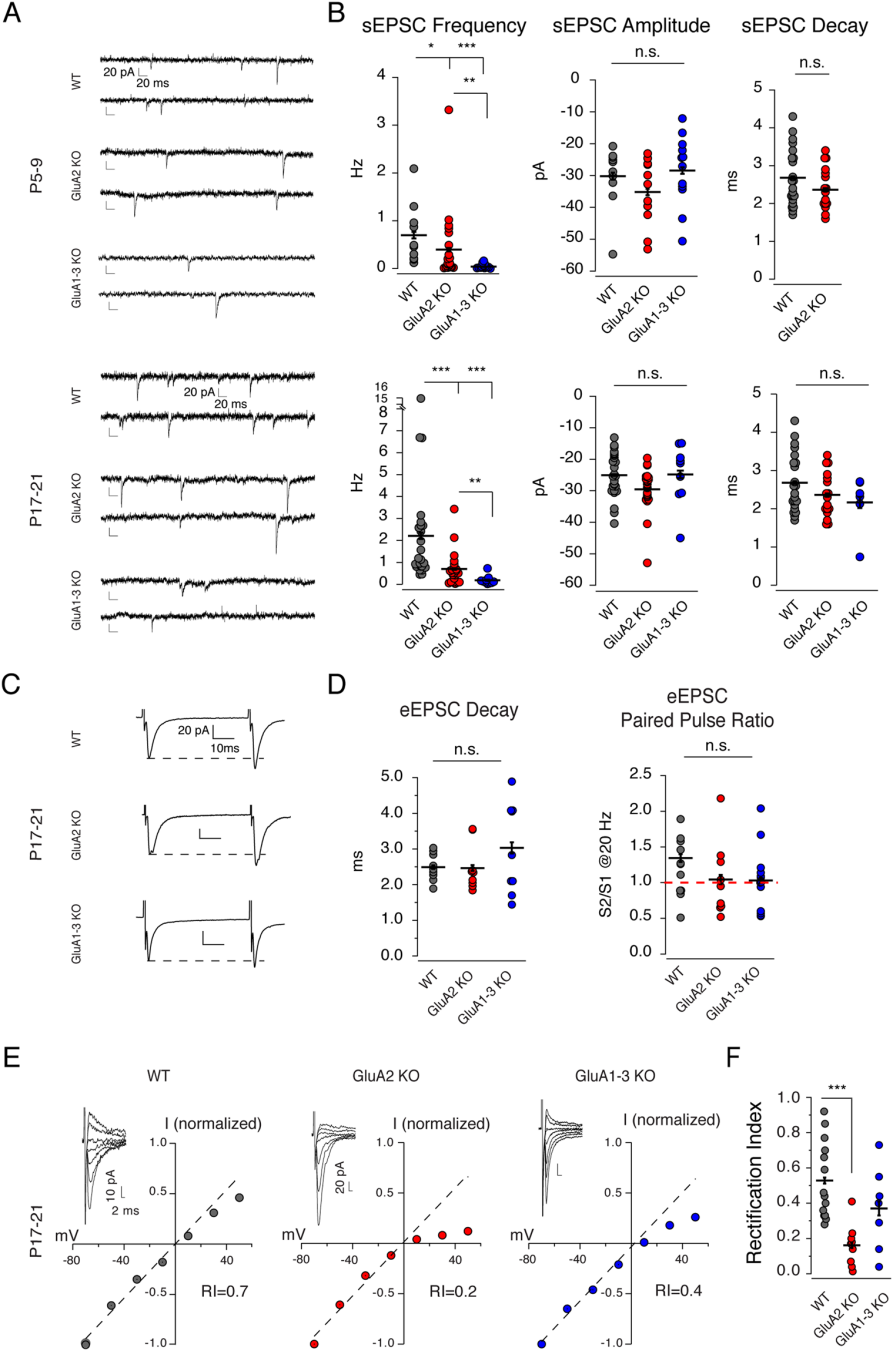
AMPAR subunit loss decreases the frequency of sEPSCs (**A**,**B**). (**A**) Representative traces of AMPAR-mediated spontaneous currents from individual neonatal (P5-9) or juvenile (P17-21) tdTomato expressing CGE-derived interneurons in stratum radiatum of CA1 hippocampus. sEPSCs in CGE GluA1-3 KO mice were rare and detected at less than 0.2 Hz (at P17-21) in the entire time window but are overrepresented in the image to allow inspection of residual AMPAR-mediated synaptic activity in the triple KO. (**B**) Scatter graphs of sEPSC frequency, amplitude and decay summarizes the group data. Both GluA2 KO and GluA1-3 KO in CGE interneurons significantly reduced the frequency of sEPSCs. sEPSC amplitude is slightly reduced upon GluA2 subunit loss at both time points, however, not significant. Similarly, sEPSC decay is slightly but not significantly reduced in KO groups. Note that not enough number of events were detected for P5-9 GluA1-3 KO group to reliably calculate the average sEPSC decay for this group. (**C**) Representative AMPAR-mediated evoked EPSC traces from individual CGE-derived interneurons located in the CA1 stratum radiatum. Dashed lines demarcate the peak of the first EPSC for reference. All three genotypes show facilitating short-term synaptic plasticity when paired pulses are evoked at 20 Hz. (**D**) Neither the decay time constant nor the paired pulse ratio is altered following elimination of either GluA2 or GluA1-3. (**E**) Representative current-voltage relationships of evoked AMPAR-mediated EPSCs. (**F**) Plots of the rectification indices for the group data. WT eEPSCs possessed an essentially linear I-V relationship. Elimination of the GluA2 subunit resulted in an inwardly rectifying I-V relation and a weakly rectifying I-V relationship following elimination of the GluA1-3 subunits. The data were compared for significance with ANOVA and a *posthoc* Wilcoxon-Mann-Whitney Test. *p < 0.05; **p < 0.005; ***p < 0.0005.

To validate the alteration of AMPARs in the CGE-derived interneuron cohort of both knockout mouse lines we first recorded spontaneous EPSCs (sEPSCs) from CGE-derived interneurons located in the stratum radiatum (sr) of CA1 hippocampus from WT, GluA2 KO, and GluA1-3 KO animals over two developmental time points (neonatal: P5-9, juvenile: P17-21) (Fig. 1). sEPSC frequency was significantly reduced in the GluA2 KO (P5-9 WT 0.7 ± 0.2 Hz versus 0.4 ± 0.08, n = 10 and 22 respectively), and almost completely eliminated in the GluA1-3 KO (P5-9 0.04 ± 0.01 Hz, n = 20) (Fig. 1B and Table 1). Despite a reduction in event frequency, the amplitudes and decay time constants of events that remained in both mouse lines were largely unchanged compared to WT (Fig. 1B and Table 1). In WT, during the first two postnatal weeks the frequency of sEPSCs increased approximately 3-fold to reach an upper limit of approximately 2 Hz (0.7 Hz at P5–9 to 2.18 Hz at P17–21, Table 1). Although an increased frequency of sEPSCs was observed in both the GluA2 KO (0.7 ± 0.08 Hz, n = 22) and the GluA1-3 KO (0.15 ± 0.05, n = 13 Hz) across the same developmental time points, their maximal frequencies obtained fell far short of that seen in WT (Fig. 1B; Table 1).

**Table 1:** Properties of glutamatergic synapses on WT, GluA2 KO and GluA1-3 KO CGE interneurons.

	P5-9			P17-21		
	WT	GluA2 KO	GluA1-3 KO	WT	GluA2 KO	GluA1-3 KO
*sEPSC*						
Frequency (Hz)	0.70 ± 0.20	0.40 ± 0.08* *p = 0.014	0.04 ± 0.01***^,$$^ ***p = 0.03 × 10^−5^ ^$$^p = 0.013	2.18 ± 0.03	0.70 ± 0.08*** ***p = 0.04 × 10^−2^	0.15 ± 0.05***^,$$^ ***p = 0.03 × 10^−7^ ^$$^p = 0.0017
n	10	22	20	22	22	13
Amplitude (pA)	−30.21 ± 3.07	−35.0 ± 1.11	−28.43 ± 0.01	−25.08 ± 0.62	−29.5 ± 0.74	−24.82 ± 3.19
n	10	22	12	22	22	9
Decay tau (ms)	2.12 ± 0.29	1.94 ± 0.07	N/A	2.78 ± 0.09	2.4 ± 0.07	2.17 ± 0.15
n	6	9		19	17	6
*AMPAR-med eEPSC*						
Decay tau (ms)	N/A	N/A	N/A	2.73 ± 0.11	2.47 ± 0.22	3.18 ± 0.32
n				10	9	10
PPR	1.52 ± 0.12	1.63 ± 0.20	1.71 ± 0.23	1.31 ± 0.18	1.04 ± 0.17	1.07 ± 0.09
n	26	5	7	14	9	18
RI	N/A	N/A	N/A	0.53 ± 0.06	0.16 ± 0.04*** ***p = 0.07 × 10^−3^	0.35 ± 0.08
n				14	9	8
*NMDA/AMPA ratio*	0.84 ± 0.07	0.99 ± 0.26	5.41 ± 3.86* *p = 0.024	0.77 ± 0.02	0.77 ± 0.02	2.92 ± 0.81***^,$$$^ ***p = 0.05 × 10^−4^ ^$$$^p = 0.03 × 10^−3^
n	19	8	8	24	18	18
*NMDA-med eEPSC*						
Decay tau (ms)	212.7 ± 15.5	199.8 ± 18.1	194.5 ± 10.1	162.9 ± 12.4	179.5 ± 17.4	179.6 ± 16.8
n	17	17	20	31	19	27
Ifenprodil/baseline (%)	39 ± 5	41 ± 4	36 ± 5	56 ± 6	56 ± 11	58 ± 6
n	8	8	5	15	6	11

Values shown as mean ± SEM. (*p < 0.05, **p < 0.005, ***p < 0.0005; ^$^p < 0.05, ^$$^p < 0.005, ^$$$^p < 0.0005).

*Indicates the comparison between KO and WT.

^$^Indicates the comparison between GluA1-3 KO and GluA2 KO.

Note.Even though sparse sEPSCs were monitored on GluA1-3 KO CGE interneurons in P5-9 age group, the number of events collected were not enough to calculate the decay tau of the current waveforms. Therefore, that information is N/A in P5-9, GluA1-3 KO group in the table.

### Physiological properties of evoked AMPA mediated EPSCs following GluA subunit elimination

In a previous study^10^, we demonstrated that elimination of NMDARs on CGE-derived hippocampal NGFCs of the CA1 stratum lacunosum-moleculare altered the properties of both pre- and postsynaptic transmission. Therefore, we next investigated the consequences of altering AMPAR composition on Schaffer collateral (SC)-mediated synaptic transmission onto CGE-derived CA1 hippocampal interneurons (Fig. 1C–F). Although we could routinely evoke synaptic events in the GluA2 KO, it was often extremely difficult to reliably evoke synaptic currents in the GluA1-3 KO and higher stimulation intensities were occasionally employed in these recordings. On many occasions despite the lack of AMPAR-mediated EPSCs in GluA1-3 KO interneurons we could observe NMDAR-mediated EPSCs which suggests the existence of “silent synapses” on these interneurons. Neither the decay time constant, nor the paired pulse ratio (measured at 20 Hz) of EPSCs measured in the GluA2 KO were different from WT (Fig. 1D; Table 1). Similarly, when it was possible to evoke EPSCs in GluA1-3 KOs their decay time constant and paired pulse ratio was similar to WT (Fig. 1D; Table 1). SC-evoked EPSCs onto WT CGE-derived CA1 interneurons possess a linear current voltage (I-V) relationship^26^ (Fig. 1E; Table 1). Elimination of GluA2 resulted in an inwardly rectifying I-V phenotype, as would be expected for GluA2-lacking Ca^2+^-permeable AMPAR-mediated events (Fig. 1E)^7,18^. In contrast, the I-V relationship of the residual currents in GluA1-3 KOs was essentially linear or weakly rectifying (Fig. 1E,F; Table 1), suggesting that the AMPARs that remain at these synapses share properties similar but not identical to WT receptors. AMPAR-mediated events that remain may result from incomplete or slow elimination of the GluA1-3 subunits or compensation by GluA4 subunits.

### Loss of GluA1-3 differentially alters survival of specific cohorts of CGE-derived interneurons

**Figure 2:**
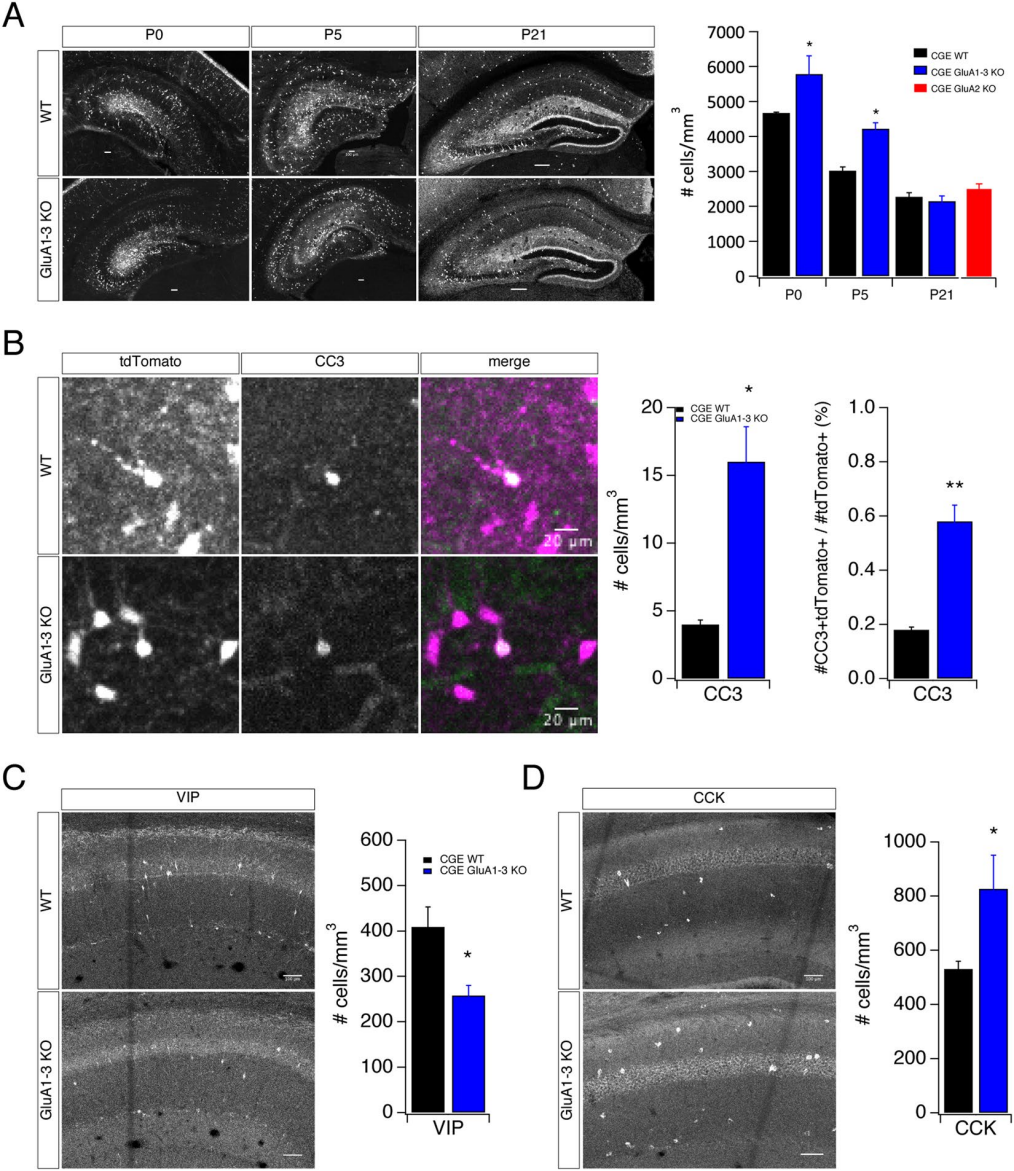
Decreased AMPAR-mediated transmission increases interneuron hippocampal invasion and differentially influences VIP- and CCK-containing interneuron survival. (**A**) Confocal images of hippocampi from WT and GluA1-3 KO mice at P0, P5 and P21. tdTomato fluorescent signal was used as a proxy to identify 5HT3AR-expressing CGE-derived interneurons. Fluorescent cells populate all layers of CA1 in hippocampus together with CA3 and dentate gyrus (DG) regions. Bar graph of cell density quantification performed on CA1 regions on mouse hippocampi. (**B**) Confocal images of hippocampi from WT (top row) and GluA1-3 KO (bottom row) mice at P5 show representative tdTomato expressing (left) CGE interneuron soma immunopositive for cleaved caspase 3 (CC3) (middle). Colocalization of tdTomato and CC3 signals are shown in the merged images (right). Bar graphs of cell quantification performed for CC3 positive CGE-derived interneurons shown as the density of CC3+ tdTomato+ cells and the fraction (%) of CC3+ cells in tdTomato+ population in the entire hippocampi. (**C**,**D**) Confocal images of hippocampi from WT and GluA1-3 KO mice at P20-P22 immunolabeled against VIP (**C**) and CCK (**D**) interneuron markers. Bar graphs of cell quantification performed on CA1 hippocampus for interneuron subgroups. *p < 0.05; **p < 0.005.

During development, migrating CGE-derived interneurons enter the hippocampus at ~E15 with a peak invasion occurring around E18.5^6^. Between their peak at E18.5 and P10 CGE-derived cells exhibit a dramatic reduction (~80% reduction) in cell density^6^ driven by apoptosis^15^. AMPARs are often expressed at embryonic stages, prior to the establishment of any synaptic contact^27–29^, suggesting potential nonsynaptic roles for AMPARs in cellular migration and survival^1,7,14,15^. To determine whether AMPARs have a regulatory or permissive role in cell migration and survival during development we counted CGE-derived interneurons in WT, GluA2 KO and GluA1-3 KO between P0 and P21 (Fig. 2A). At P21 the total surviving CGE-derived interneurons in WT, GluA2KO and GluA1-3 KOs was indistinguishable. However, at early postnatal stages (P0 and P5) cell invasion of CGE-derived interneurons into the nascent hippocampus of the GluA1-3 KO exceeds that of WT (Fig. 2A). Since the numbers of CGE-derived interneurons ultimately converge in P21 WT and the GluA1-3 KO we next determined whether increased programmed cell death accounted for this marked reduction of cells in the GluA1-3 KO. Using an antibody against cleaved caspase 3 (CC3), an apoptotic cell death marker, we observed an increased incidence of CC3-labeled CGE-derived interneurons in the GluA1-3 KO compared to WT (Fig. 2B). Previous studies have suggested that the survival of different interneuron subpopulations is selectively vulnerable to changes in ongoing activity^15,30,31^.

Priya, *et al*.^15^ recently demonstrated that ongoing activity was essential for cortical CGE-derived interneuron survival and that overexpression of the inward rectifying potassium conductance, Kir2.1, to dampen excitability resulted in an ~10% reduction in cell numbers. Of interest, cortical VIP-containing interneuron numbers were resistant to changes in ongoing activity. Therefore, we investigated whether elimination of AMPARs early in development similarly impacts the survival of either CCK- or VIP- subpopulations of CGE-derived interneurons. Immunolabelling revealed that although the overall cell density of CGE-derived interneurons was unchanged in the GluA1-3 KO (Fig. 2A) the number of VIP-positive interneurons was significantly reduced, while the number of CCK-containing interneurons increased (Fig. 2C,D). Taken together these data suggest that elimination of AMPARs initially increases cell invasion into the hippocampus during early postnatal development, however due to increased apoptosis overall cell numbers ultimately consolidate to that observed in WT. However, loss of GluA1-3 alters the ratio of surviving VIP-:CCK-containing interneurons and unlike CGE-derived interneurons of the cortex, both CCK- and VIP-containing interneuron populations are sensitive to activity levels.

### The GluA2 subunit is a key determinant of glutamatergic synapse number

**Figure 3:**
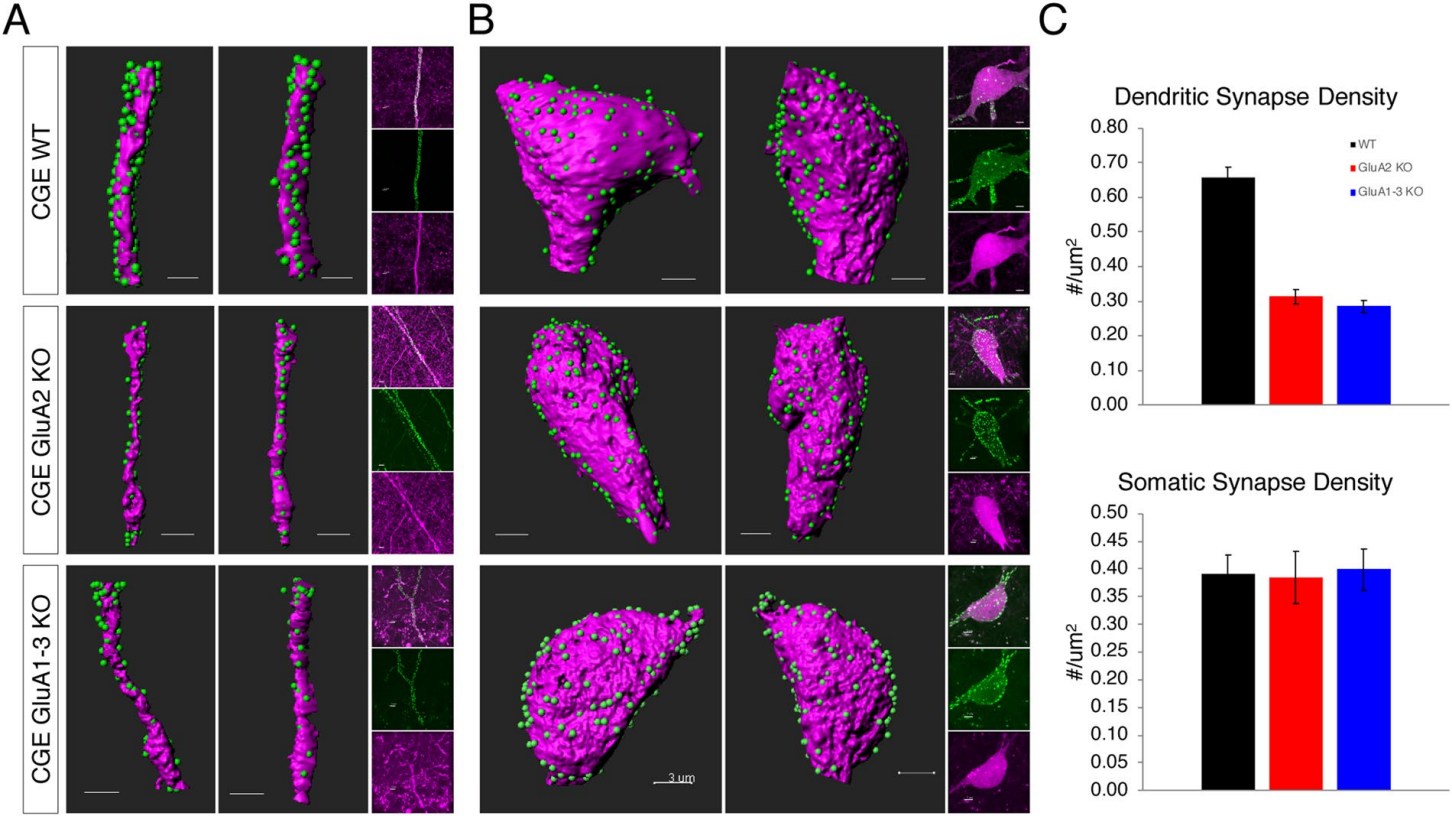
The density of dendritic but not somatic glutamatergic synapses is determined by the presence or absence of GluA2. (**A**,**B**) Images show (from two different angles) Airyscan (LSM) super-resolution images of green fluorescent puncta (PSD-95_EGFP) detected on magenta fluorescent (tdTomato) dendrites and somata converted using Imaris software (Bitplane). PSD-95_EGFP expression that created a punctate signal on the dendrites and soma, is used as a proxy for glutamatergic synaptic sites in 3D space. The tdTomato signal was used to create a surface for dendrites and soma and the EGFP signal that did not fall within 1 µm distance from the created surface was first automatically and then, if necessary, manually eliminated from the analysis. Scale bars, 3 µm. small panels to the right represent the raw images used for analysis. (**C**) Bar graphs of the density of synaptic sites on dendrites (WT, n = 64; GluA2 KO, n = 30; GluA1-3 KO, n = 34) or soma (WT, n = 27; GluA2 KO, n = 22; GluA1-3 KO, n = 13) of the CGE interneurons. The data for all three groups were compared for significance with ANOVA and a *posthoc* Wilcoxon-Mann-Whitney Test when a significance was found with ANOVA. The density of synaptic sites on soma are similar in all groups compared. The density of synaptic sites is significantly reduced on KO CGE interneurons compared to WT: GluA2 KO to WT, p = 0.034 × 10^−8^, GluA1-3 KO to WT, p = 0.024 × 10^−8^, GluA1-3 to GluA2, p = 0.53. ***p < 0.0005.

Our observation of a reduced frequency of synaptic activity in both GluA2, and GluA1-3 KOs suggests that the loss or reduction of AMPA subunits may determine the number of synaptic contacts made onto these cells. To test this hypothesis, we injected an adeno-associated virus (AAV) that harboured a recombinant PSD-95-EGFP coding sequence into both WT and KO mouse hippocampus. The expression of PSD-95-EGFP was driven by Cre expression under the 5HT3AR promoter in all lines. Approximately 2 weeks after injection, tissue was collected (P36-37) and PSD-95-EGFP puncta were analysed as a proxy for glutamatergic synaptic sites on both somatic and dendritic compartments, predominantly within the sr, of randomly chosen individual CGE-derived interneurons. The density of fluorescent puncta on dendritic surfaces of GluA2 KO CGE interneurons was significantly reduced compared to WT (Fig. 3A–C). In contrast, the density of fluorescent puncta on somatic sites was unchanged. Of interest, synapse density was not further reduced in the GluA1-3 KO compared to the GluA2 KO, suggesting that the presence or absence of the GluA2 subunit alone is a major determinant of dendritic synapse density.

### Excitatory input is a major determinant of dendritic arbour

The anatomical features of interneuron subpopulations are typically distinct, with specific rules for dendritic and axonal arbours that are determined by both genetic factors and activity^6,7,32^. Glutamate receptor expression profiles have been linked to cell-specific interneuron anatomical, synapse and circuit rearrangements^10,24,25,33,34^. For example, a loss of NMDARs altered glutamate synapse development^34^ and reduced the dendritic complexity of somatosensory CGE-derived reelin-positive NGFC cells, but failed to alter VIP-interneuron morphology^24^. In contrast, loss of NMDARs in hippocampal CGE-derived NGFCs caused dendritic *hypertrophy* and altered the somato-dendritic polarity^10^. Whether loss of AMPAR subunits results in similar anatomical deficits is unclear.

**Figure 4:**
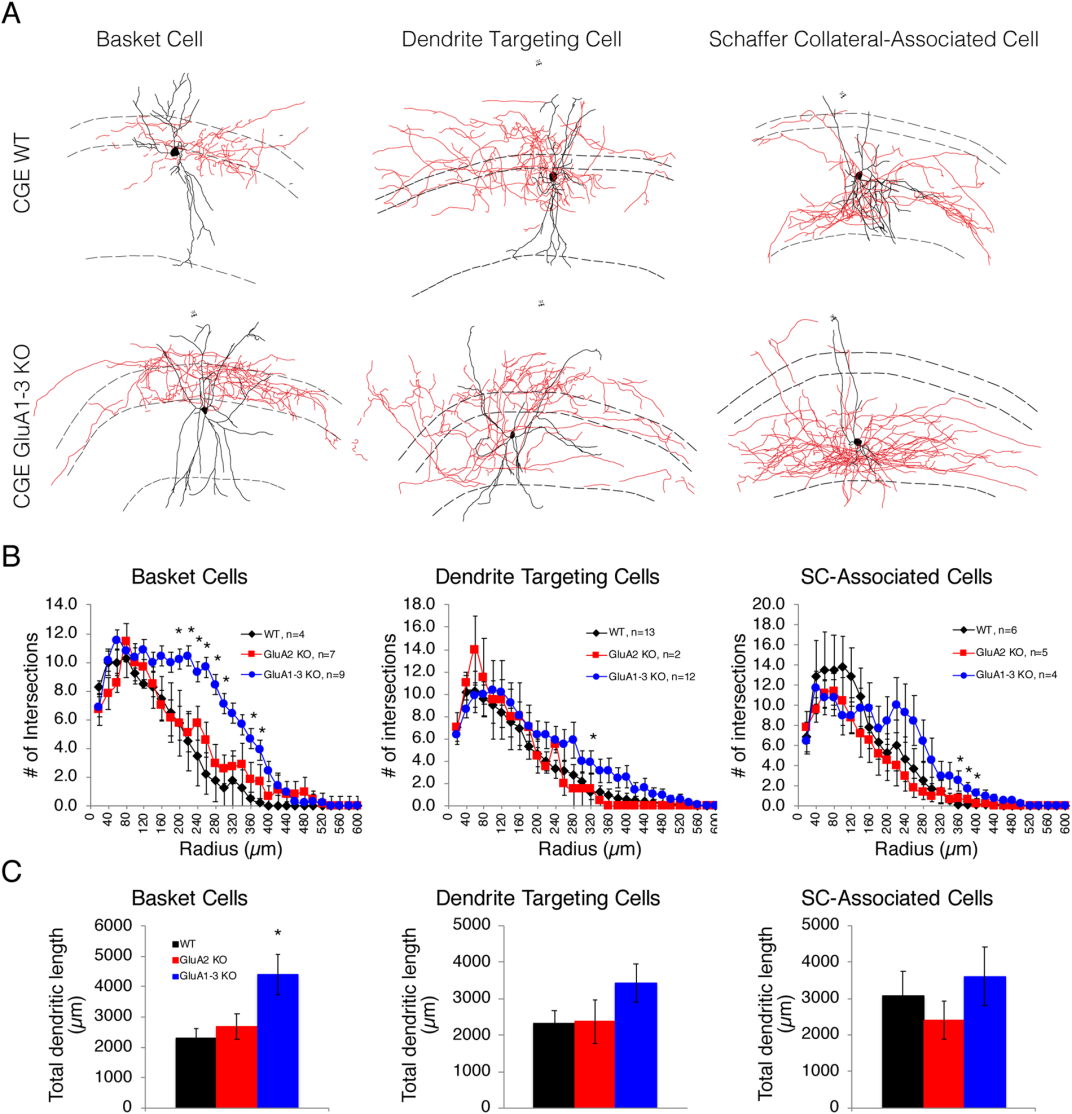
GluA1-3 KO CGE interneurons have more extensive dendritic arbours. (**A**) Representative samples of reconstructed WT or GluA1-3 KO CGE basket, Wide arbour dendrite targeting, and Schaffer collateral associated cells in CA1 hippocampus. Dendrites (black) and axons (red) were defined based on the fluorescent signal and the apparent differences in their thickness. Dendrites of all three morphological subgroup extend to stratum oriens, stratum pyramidale, stratum radiatum and sometimes stratum lacunosum-moleculare layers of CA1. Dashed lines demarcate the borders of these layers. (**B**) Line graphs for dendritic branching depicted as number of intersections on concentric circles (20 μm intervals) from the cell soma by Scholl analysis. (**C**) Bar graphs for the total dendritic length indicate that only basket cells increased their overall dendritic length. Significance tested with ANOVA and a *posthoc* Tukey Test. *p < 0.05.

Basket-, Wide-arbour dendrite targeting-, and SC associated-interneurons are three major morphologically identifiable CGE-derived interneuron subtypes found in the CA1 stratum pyramidale and radiatum (Fig. 4A)^6,26^. Using reconstructions of biocytin-filled neurons, we performed Scholl analysis of the dendritic profiles of these subpopulations in WT, GluA2 and GluA1-3 KOs (Fig. 4). Elimination of the GluA2 subunit alone to render AMPARs Ca^2+^-permeable had little, to no, impact on the dendritic complexity. In contrast, in all three cell types loss of GluA1-3 caused dendritic hypertrophy (Fig. 4A–C). The basket cell dendrites were most impacted by loss of GluA1-3, with an increased number of branch points together with an increased overall dendritic length. In contrast, while neither Wide-arbour dendrite targeting cells, nor SC-associated cells showed an overall increase in dendritic length, both possessed a significant increase in the number of dendritic branch points. These data suggest that the reduction in synapse density shown above (Fig. 3) does not simply result from a dilution of synapse number by an increased dendritic arbour since elimination of GluA2 alone reduces synapse number (Fig. 3) without altering the dendritic profile (Fig. 4B). The hypertrophy of dendrites in the GluA1-3 KO is likely a result of the near complete loss of excitatory input onto these cells triggering an expansion of the dendritic receptive area to compensate for this synaptic input loss, underscoring the role for excitatory input in determining cell arbour complexity.

### NMDAR subunit composition in CGE interneurons is independent of AMPARs

**Figure 5:**
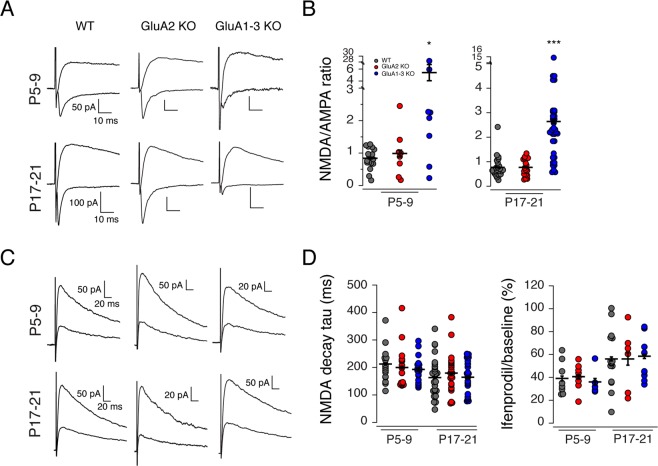
Changes in AMPAR subunit composition does not alter the properties of NMDARs. (**A**) Outward Schaffer collateral evoked NMDAR-mediated EPSCs (V_hold_ = +50 mV, in DNQX) and inward AMPAR-mediated EPSCs (V_hold_ = −70 mV, in Mg^2+^) recorded from WT, GluA2 KO, and GluA1-3 KO CGE interneurons in P5-9 and P17–21 mice. As early as P5, all interneurons exhibited both AMPA and NMDAR-mediated evoked activity. NMDAR-mediated EPSCs were evoked at a reasonably fixed amplitude of ~100–150 pA revealing only a small corresponding AMPAR-mediated component in CGE GluA1-3 KO interneurons. (**B**) Scatter plots of NMDA:AMPA ratios for all three mouse lines at two different developmental time points. Elimination of GluA1-3 subunits but not the GluA2 subunit increases the NMDA:AMPA EPSC ratio largely through a reduction in the corresponding AMPAR-mediated EPSC. (**C**) NMDAR-mediated EPSCs (V_hold_ = +50 mV) in the presence (smaller traces) and absence (larger traces) of the GluN2B-expressing NMDAR blocker ifenprodil, recorded from WT, GluA2 KO and GluA1-3 KO CGE interneurons from P5-9 and P17-21 mice. (**D**) Scatter plots of the NMDAR-mediated decay tau of NMDAR-mediated EPSCs and the magnitude of ifenprodil block of the NMDAR-mediated EPSC. The data for all three groups were compared for significance with ANOVA and a *posthoc* Wilcoxon-Mann-Whitney Test when a significance was found with ANOVA. *p < 0.05; ***p < 0.0005.

At SC synapses onto WT CGE-derived interneurons, GluA2-containing, Ca^2+^-impermeable AMPARs are typically found in association with GluN2B-containing NMDARs^26^. It is unclear whether the type of NMDARs present at these synapses is dictated or influenced by the composition of its corresponding AMPARs. To probe the relationship between synaptic AMPAR and NMDARs we evoked synaptic responses in both WT, GluA2-KO and GluA1-3 KO interneurons across two developmental ranges (neonatal P5-9 and juvenile P17-21). The NMDAR/AMPAR ratio was extracted by evoking NMDAR-mediated EPSCs (V_hold_ = +50 mV) of a relatively fixed amplitude (100–150 pA) and then obtaining the corresponding AMPAR mediated component at −70 mV. The NMDAR/AMPAR ratio was comparable in recordings from WT and GluA2 KO but was significantly greater in recordings from GluA1-3 KOs consistent with a near loss of all synaptic AMPARs at these synapses (Fig. 5A,B; Table 1).

At SC synapses onto MGE-derived interneurons there is a developmental switch in NMDARs such that initial GluN2B-containing receptors are replaced with GluN2A-containing receptors during postnatal development. This “2B-to-2A” switch is governed in part by Ca^2+^ entry through GluA2-lacking, Ca^2+^-permeable AMPARs at these synapses^26^. This 2B-to-2A switch endows NMDARs with more rapid kinetics and a reduced pharmacological sensitivity to the GluN2B-containing NMDAR antagonist, ifenprodil. CGE-derived interneurons lack this developmental switch and express GluN2B-containing NMDARs across all age ranges tested^18,26^. We next wanted to determine whether altering the AMPAR composition, and its Ca^2+^-permeability directly influenced the nature and composition of their corresponding synaptic NMDARs. Surprisingly elimination of either GluA2 or GluA1-3 was without impact on the evoked NMDAR decay kinetics (Fig. 5C,D; Table 1) which remained comparable across the two age ranges tested, despite changes in AMPAR composition and number. Similarly, the magnitude of block by the selective GluN2B-containing receptor antagonist, ifenprodil, was unchanged across all three genotypes indicating that GluN2B expression profile was also unaltered by elimination of either GluA2 or GluA1-3 (Fig. 5D; Table 1). These data indicate that NMDAR subunits trafficked to and clustered at synaptic sites are independent of the existing AMPAR pool at these synapses.

### AMPAR loss on CGE-interneurons impairs hippocampal network function

**Figure 6:**
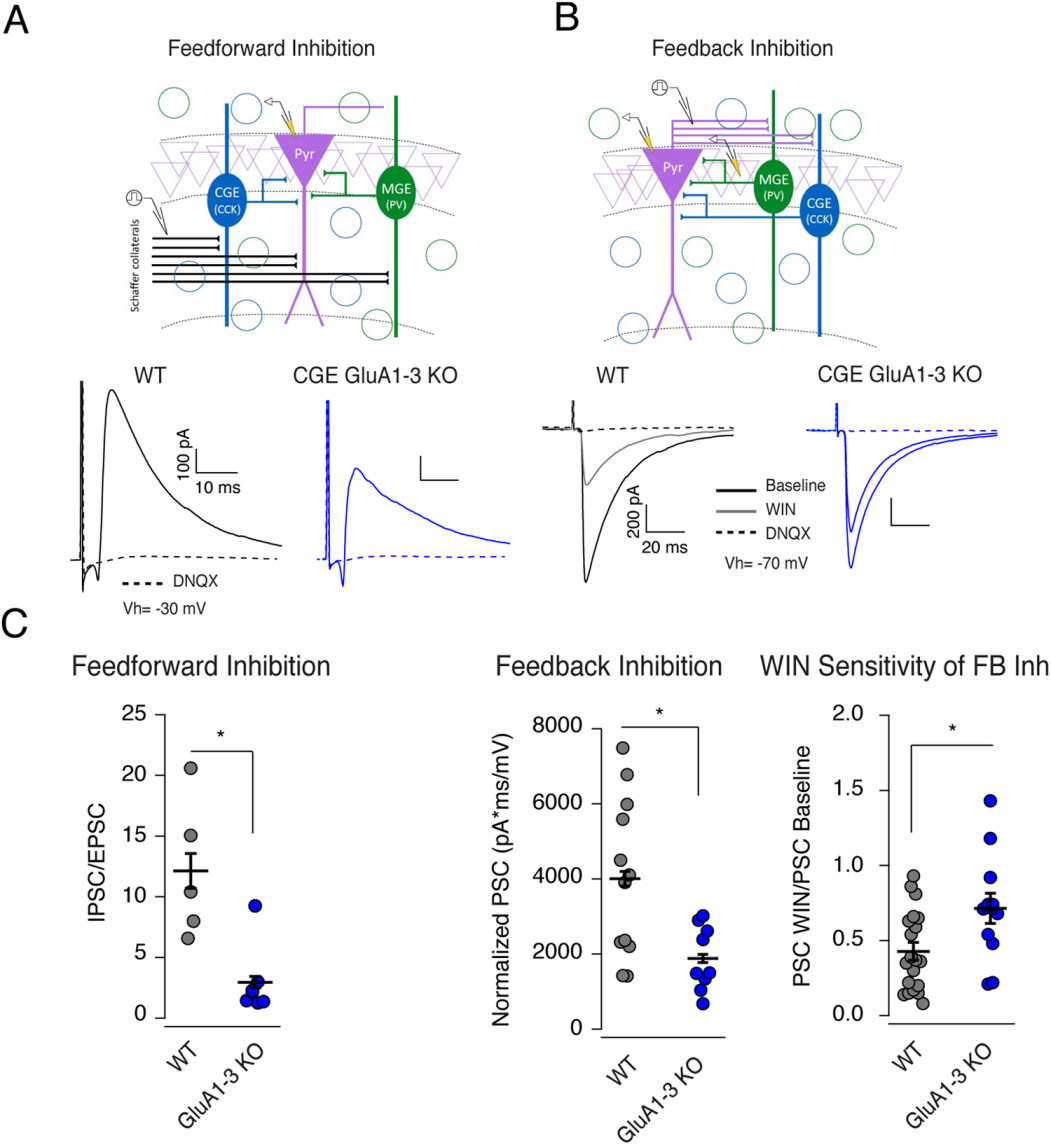
Elimination of AMPAR subunits diminishes the role of CGE interneurons in both feedforward and feedback inhibition onto CA1 pyramidal neurons. (**A**) Upper: Schematic of canonical feedforward inhibitory circuit within the CA1 circuit together with electrode placement. Lower: Evoked monosynaptic EPSCs (inward current) and disynaptic IPSCs (outward current) recorded at V_hold_ = −30 mV from CA1 pyramidal cells in WT (black traces), and CGE GluA1-3 KO (blue traces). Dashed traces show complete elimination of both mono- and disynaptic evoked events by DNQX and APV. (**B**) Schematic of canonical feedback inhibitory circuit onto CA1 pyramidal cell together with stimulating and recording electrode placement. Lower traces show evoked disynaptic IPSCs recorded at V_hold_ = −70 mV from CA1 pyramidal cells in WT, and CGE GluA1-3 KO under control and in the presence of the CB1 receptor agonist WIN55, 212-2. Dashed traces show residual events recorded in the presence of DNQX and APV confirming the disynaptic nature of evoked inhibition. (**C**) Group data for feedforward inhibition (left) feedback inhibition (middle) and the degree of WIN sensitivity of feedback inhibition in both WT and GluA1-3 KO. Significance of the differences between groups compared was calculated with Wilcoxon-Mann-Whitney Test. For Feedforward Inhibition (IPSC/EPSC): WT, n = 5 and GluA1-3 KO, n = 7; p = 0.01. For Feedback Inhibition (Normalized PSC): WT, n = 12 and GluA1-3 KO, n = 9; p = 0.034 and WIN Sensitivity (WIN/baseline PSC): WT, n = 20 and GluA1-3 KO, n = 13; p = 0.011. *p < 0.05.

In hippocampal microcircuits, MGE- and CGE-derived inhibitory interneurons participate in both feedforward and feedback regulation of afferent activity^35–40^. We next investigated whether the loss of AMPARs, and the consequent reduction of glutamatergic synapse density impacted the contribution of CGE-derived cells to both feedback and feedforward inhibitory drive. We made recordings from CA1 pyramidal cells and evoked monosynaptic SC-mediated EPSCs and disynaptic IPSCs to monitor feedforward inhibition in both WT and GluA1-3 KOs (Fig. 6A) (recordings from GluA2 KO were not performed since their evoked EPSCs shared properties similar to WT, Fig. 1). At a holding potential of −30 mV, we were able to simultaneously record inward EPSCs and outward IPSCs. The AMPAR-mediated component was evoked around a fixed amplitude of 20–30pA to allow comparison of the disynaptic IPSCs. Under these conditions, the I/E ratio (Fig. 6A,C) was significantly reduced (~76%) in GluA1-3 KO mice compared to WT (I/E ratio WT: 12.13 ± 0.01, n = 5; GluA1-3 KO: 2.96 ± 0.90, n = 7). At the end of the recording the di-synaptic nature of the inhibitory current was verified by pharmacological block of excitatory synaptic transmission by DNQX.

To probe the feedback inhibitory input onto CA1 pyramidal cells (Fig. 6B) we recorded from pyramidal cells while antidromically stimulating their axons in the alveus^40^. In addition, we placed an extracellular recording electrode in the pyramidal cell layer to monitor the population field EPSP, which we used to normalize the antidromic disynaptic IPSC. We found that elimination of GluA1-3 in CGE interneurons resulted in a 62% reduction of feedback inhibition onto CA1 pyramidal cells compared to WT (Fig. 6B,C) (WT: 4007 ± 607.1 pA.ms/mV, n = 12; GluA1-3 KO: 1560 ± 290.3 pA.ms/mV, n = 4). Although a number of CGE-derived inhibitory interneurons likely contribute to the total feedback inhibition, CCK-containing cells contribute a significant proportion of this inhibition^37,38,40^. Application of the CB1R agonist WIN55,212-2, which selectively reduces transmission from CCK-containing interneurons reduced feedback inhibition in WT by 57% but only by 29% in GluA1-3 KOs (Fig. 6C). These data suggest that despite an apparent increase in CCK-containing interneuron density following GluA1-3 elimination, their role in network activity diminishes.

### AMPAR subunit loss in CGE interneurons result in abnormal behaviour in mice

**Figure 7:**
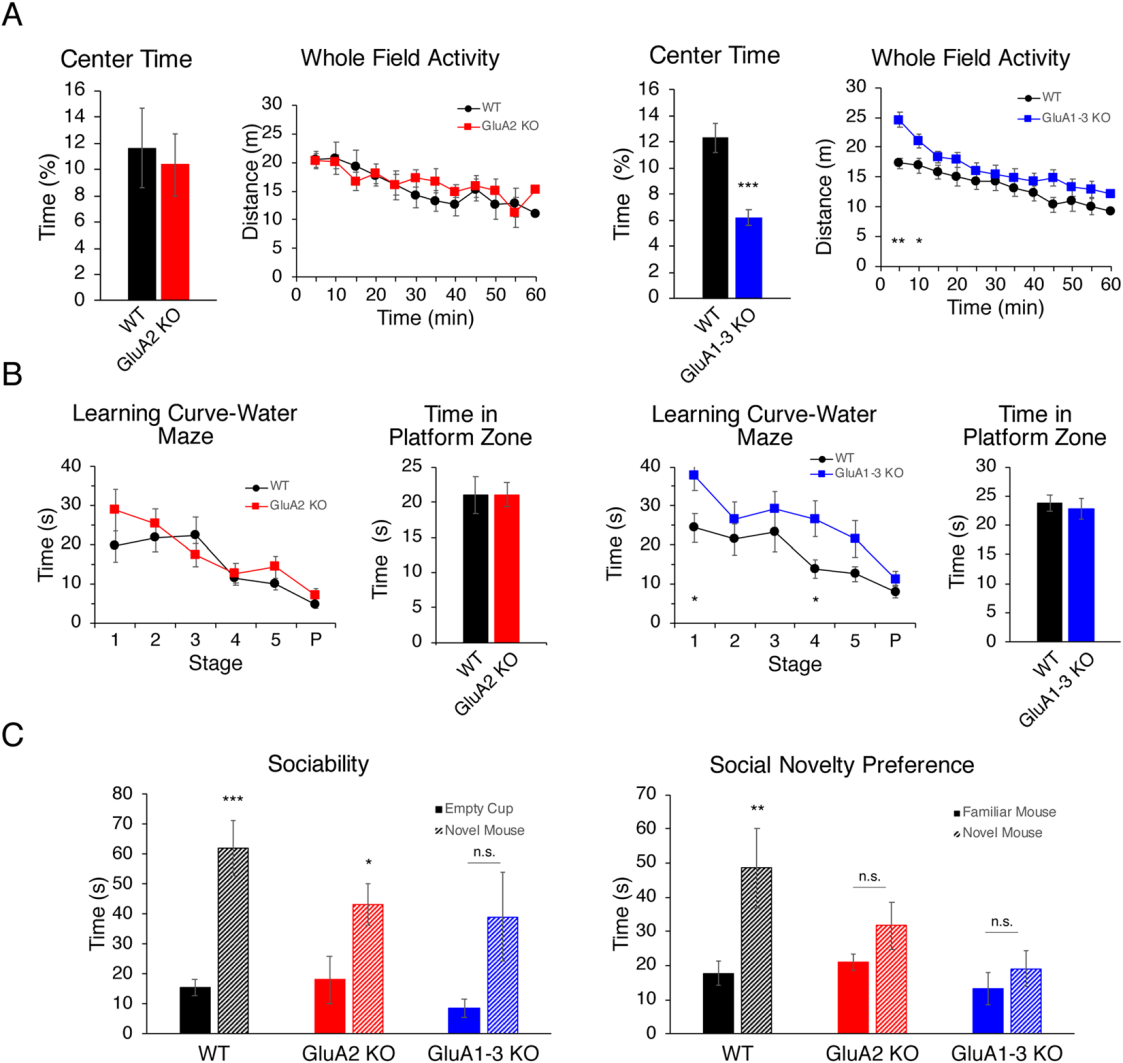
Behavioural characterization of adult CGE GluA2 KO and CGE GluA1-3 KO mice. (**A**) Group data show the explorative activity of control (black), CGE GluA2 KO (red), and CGE GluA1-3 KO (blue) mice in the whole field over 60 minutes, analysed and graphed at 5 min time segments. GluA2 KO (n = 9) vs WT (n = 7). Bar graphs represent explorative activity in the centre region over the first 5 min of the open field maze. CGE GluA2 KO mice show similar locomotor activity to WT. In contrast CGE GluA1-3 KO mice exhibit novelty induced hyperlocomotion in the first 10 minutes which then converges with WT activity during the next 50 minutes. These hyperactive GluA1-3 KO mice also avoid the centre of the open field maze during the first 5 min segment compared to control indicating a potential anxiety-like phenotype, p < 0.00002. (**B**) Group data show the learning curve (line graphs) for WT and KO mice to find the hidden platform in the Morris water maze (MWM) during 5 sequential days of learning together with the probe test (P). Bar graphs represent the time spent in the platform zone during the probe test. There is no behavioural difference between CGE GluA2 KO mice (n = 9) and WT (n = 5) during MWM. CGE GluA1-3 KO mice (n = 12) compared to WT (n = 13) show slower performance during the 5 days of training. (**C**) Group data showing the total time mice from each genotype spent interacting with a novel mouse vs an object (sociability) and interacting with a novel vs the familiar mouse (social novelty preference). All experimental (GluA2 KO, n = 9; GluA1-3 KO, n = 11) and control groups (n = 24) spent more time with a mouse of the same gender: GluA2 KO, p = 0.03; GluA1-3 KO, p = 0.058; WT, p = 0.00001. Compared to control both GluA2 and GluA1-3 KO mice did not spend significantly more time with a novel social interactor: GluA2 KO, p = 0.17; GluA1-3 KO, p = 0.41; WT, p = 0.004. *p < 0.05; **p < 0.005; ***p < 0.0005.

To characterize the behavioural phenotype(s) associated with the loss of either GluA2 and GluA1-3 in CGE-derived inhibitory interneurons, we next ran a battery of tests to assess exploratory behaviour, hippocampus-dependent learning and memory, and social memory. In the novel open field test GluA2 KO mice (n = 9) showed similar exploratory behaviour compared to WT controls (n = 7) as well as time spent in the centre of the open field during the first 5 min (Fig. 7A) indicating both groups of mice have similar responses to novel environment. However, GluA1-3 KO mice (n = 23) showed novelty-induced hyperlocomotion locomotor activity in the first 0–10 min during the 1-hour free exploration in the open field, although they habituated similarly to WT (n = 19) (Fig. 7A). Interestingly, GluA1-3 KO mice spent 50% less time in the centre region of the open field during the first 5 min of free exploration compared to the controls (KO: 18.6 ± 1.8 s, 6.2 ± 0.6%; WT: 36.9 ± 3.4 s, 12.3 ± 1.1%) (Fig. 7A), suggesting that GluA1-3 KO mice exhibit higher anxiety-like behaviour compared to WT controls^41^. In the Morris water maze GluA1-3 KO mice (n = 12), unlike GluA2 KO mice (n = 9) exhibited significantly slower spatial learning skills than WT (n = 13 and n = 5 respectively) (Fig. 7B). However, similar to both WT and GluA2 KO mice, long-term memory was intact in GluA1-3 KO mice, as reflected by the latency to reach the platform area and the time spent in the Platform zone during the probe test (Fig. 7B). Finally, dysfunction in CGE-derived interneuron circuits result in deficits in social behaviour^40,42^. In a three-chamber social interaction test both GluA2 KO (n = 9) and GluA1-3 KO mice (n = 11) exhibited blunted social novelty preference compared to WT (n = 24). However, although GluA2 KO animals showed normal sociability, GluA1-3 KO mice displayed impaired sociability (Fig. 7C). It is worthwhile pointing out that GluA2 KO mice did not show increased anxiety-like behaviour in the open field maze (Fig. 7A) suggesting that the deficit in social memory of KO mice is not a consequence of anxiety.

## Discussion

5HT3AR-expressing interneurons represent the CGE-derived inhibitory interneuron cohort that are relatively late born GABAergic interneurons^6,43^. These cells reach a peak of hippocampal invasion around E18.5 and cell numbers are then reduced and consolidated during the first two weeks postnatally as they integrate into the nascent cortical circuitry. Although the synaptic properties of glutamate receptors onto interneurons have been studied in detail^7,18,26^, little is known about the synaptic or non-synaptic role(s) played by glutamate receptors on CGE-derived interneurons during development. Here, using two mouse lines that harbour either an elimination of the GluA2 AMPAR subunit or GluA1, 2, & 3 AMPAR subunits early in the development of CGE-derived inhibitory interneurons we have uncovered a number of roles played by AMPARs in cell migration and maturation, synapse and circuit integration coupled to significant changes in behaviour.

With the exception of the GluA4 subunit, whose expression commences during the first weeks of postnatal life, and whose expression is largely limited to synapses onto MGE-derived PV-containing interneuron populations^44^, the remaining GluA1, 2 & 3 subunits are expressed in the embryonic mouse brain^27,44,45^. Although these subunits will ultimately populate excitatory synapses onto interneurons, early in their development it is highly likely that they play a non-synaptic role to influence migration and maturation of the nascent cell population^1^ since glutamate serves as a chemoattractant for cellular migration^46^. Subsequently once cells establish their final location in the hippocampus excitatory synapses are formed and typically become functional in the first two weeks of postnatal life^18,26^. Excitatory synapses onto CGE-derived interneurons typically comprise GluA2-containing Ca^2+^-impermeable AMPA receptors^18,26^. Elimination of GluA2 converted these receptors to inwardly rectifying GluA2-lacking Ca^2+^-permeable AMPARs, which was coupled to a reduction of spontaneous excitatory synaptic frequency likely due to the observed reduction in the density of PSD-95 containing dendritic glutamatergic synapses. A role for the GluA2 subunit in regulating synaptic structure and number was previously suggested^47,48^ (but see^49^). Indeed, overexpression of GluA2 *increases* spine density in hippocampal neurons and initiates spine-like formations on natively aspiny dendrites^47^. Furthermore, a disruption of the GluA2 and glyceraldehyde 3-phosphate dehydrogenase (GAPDH) interaction, decreases spine density in neuronal cultures^48^. Interestingly, the change in synapse density was observed at dendritic but not at somatic sites and suggests a differential role for GluA2 in controlling synapse number across subcellular compartments. Consistent with these anatomical changes an ~ 70% reduction in sEPSC frequency was observed in the GluA2 KO. In contrast, the observation that the paired pulse ratios remained the same suggest that the overall change in synaptic activity is largely determined by postsynaptic changes with little change in presynaptic properties. Although further elimination of the GluA1 and GluA3 subunits from CGE-derived interneurons resulted in an almost complete elimination of spontaneous synaptic activity, this surprisingly failed to further reduce synapse density. These data suggest that the presence or absence of the GluA2 subunit alone is a key determinant of synapse number on the developing CGE-interneurons. In the GluA1-3 KO, the AMPAR-mediated events that remain may reflect several possibilities that include a slow or incomplete elimination of GluA1-3 or residual GluA4 subunit activity.

Although GluA2 plays a pivotal role in determining synapse number the presence or absence of the subunit appears to have little influence over the complexity of the cell morphology. In contrast, while the elimination of GluA1-3 resulted in no further change in synapse density, elimination of functional AMPA synapses increased the overall complexity and length of dendritic branching. The role for glutamate receptors in influencing interneuron cell morphology appears complex. In reelin-positive neurogliaform cells of the hippocampal stratum lacunosum-moleculare elimination of NMDARs alters dendritic branching such that dendrites in the target zone for entorhinal cortex afferents show greater complexity whereas those targeted by SC inputs show a reduced elaboration^10^. In contrast, in somatosensory cortex a decrease in NMDAR activity correlates with decreased dendritic branching in RE-positive, but not VIP-positive, interneurons^24,30^. Taken together these data suggest that complex and often differing rules dictate the relationship between activity and morphology that likely depends on neuronal identity, the nature of the glutamate receptor population, brain region and type of afferent input received by the dendrites.

During development excessive numbers of inhibitory interneurons migrate into cortical and hippocampal regions^6,7,13^. During early postnatal life the overall numbers of interneurons are reduced by apoptosis^13^. In GluA1-3 KOs the loss of AMPARs resulted in an increased number of interneurons invading the hippocampus compared to WT at early postnatal stages. However, cell numbers converged with WT by P21 due to an increase in apoptosis. Both intrinsic and network electrical activity promote cortical interneuron survival by leveraging apoptosis^14–16,30,31,50,51^. Here the number of cells positive for an apoptotic marker was elevated following elimination of GluA1-3, suggesting that glutamate receptor activation titrates, via apoptotic mechanisms, the number of interneurons ultimately targeted for elimination. However, despite similar total numbers by P21, the relative ratio of CCK-containing, and VIP-containing interneurons was altered. The survival of CGE interneuron subgroups is differentially influenced by changes in activity^14,15,30^. De Marco Garcia, *et al*.^30^ and Priya, *et al*.^15^ demonstrated that cortical reelin-containing and calretinin-containing but not VIP-containing interneuron cell numbers were reduced by ~10% due to increased apoptosis when interneurons overexpressed an inward rectifying potassium channel Kir2.1. Our observations of increases in CCK-containing, and decreases in VIP-containing interneurons in the GluK1–3 KO are inconsistent with the observations of Priya, *et al*.^15^ and perhaps suggest that not all changes in cell excitability are equivalent and that the time window for regulation of apoptosis by activity is quite early and narrow for VIP interneurons and that our genetic manipulation occurred early enough to capture that impairment. In the Priya, *et al*.^15^ study, postnatally overexpressed Kir2.1 is used to hyperpolarize the interneuron resting membrane potential, renders cells intrinsically less excitable but does not necessarily change the synaptic input onto the cells. In fact, such a manipulation may act to *increase* synaptic input due to the resulting increased driving force for AMPAR-mediated transmission at more hyperpolarized potentials. Our elimination of AMPARs during embryonic days likely invokes a fundamentally different mechanism for cell survival that remains to be determined.

Our experiments investigating feedforward and feedback inhibition confirm a role for CGE-derived interneurons in both phenomenon^35,40^. The reduction of the CGE-derived inhibitory component in the GluA1-3 altered the overall ratio of MGE-derived to CGE-derived involvement in both circuit processes. Moreover, our observation that elimination of GluA1-3 alters the subtype composition of the CGE-cohort suggests that elimination of VIP-containing interneurons and the preservation of CCK-containing interneurons may be a homeostatic mechanism that compensates for changes in feedforward and feedback inhibition. However, despite the increased number of CCK-containing interneurons the amount of WIN-sensitive component in feedback inhibition was markedly *reduce*d compared to wildtype as a result of the absence of excitatory drive onto these cells. We cannot rule out that either homeostatic changes in intrinsic membrane properties or the inhibitory output of GluA1-3 KO CGE interneurons also contribute to the alterations in feedforward and feedback inhibition. Interestingly, VIP and CCK-expressing interneuron subgroups partially overlap^52^ and how each subset of nonoverlapping/overlapping VIP and CCK populations contributes to our observed VIP:CCK shift requires further investigation.

The elimination of GluAs in the developing circuit has major consequences for several behavioural tasks in adult mice. The loss of GluA1-3 in CGE-derived interneurons results in hyperlocomotion, learning deficits and impaired social behaviour in adult mice. Consistent with our observations previous studies have also indicated that disruption of CGE interneuron integration into the neuronal network results in cognitive impairments in rodents^40,42,53^. miRNA silencing of glutamic acid decarboxylase 1 (GAD1) in the CCK-containing or the NPY-containing populations led to mice with hyperlocomotion in open field maze or learning deficits, respectively^42^. Reduction in synaptic connections made by CCK-containing interneurons causes impaired exploratory behaviour and spatial learning and memory in mice^53^. Additionally, CCK population loss in hippocampal networks following maternal cannabinoid administration results in compromised feedforward and feedback inhibition in offspring that was coupled to social behavioural deficits in adulthood^40^. However, it should be pointed out that these behaviour tasks not only reflect changes in hippocampal CGE-interneuron driven activity but likely are the result of the widespread changes in AMPAR expression occurring more broadly than just the hippocampus.

In conclusion, our data demonstrate that AMPAR subunits differentially contribute to numerous aspects of the development and maturation of CGE-derived interneurons and hippocampal circuitry that are essential for normal animal behaviour.

## Methods

### Animals

Four transgenic mouse lines were used to create a conditional reporter line for CGE-derived interneurons (5HT3R-Cre:Ai14), a conditional single (GluA2 KO) and a triple (GluA1-3 KO) knockout lines: 5HT3R-Cre driver line: Htr3A BAC-Cre mouse line (founder line NO152) was obtained from C. Gerfen (NIMH, NIH), tdTomato reporter line: Ai14 (B6;129S6-*Gt(ROSA)26Sor*^*tm14(CAG-tdTomato)Hze*^/J; cat # 007914 Jackson Laboratory), AMPAR subunit knockout lines: GRIA2^fl/fl^, GRIA1–3^fl/fl^ ^54–56^. Both male and female mice were used for experiments.

### Statement

All animal studies have been approved by, and all methods were performed in accordance with the guidelines and regulations set by the National Institutes of Health’s Institutional Animal Care and Use Committee (IACUC).

### Immunohistochemistry

#### Antibodies

The following primary antibodies were used: rabbit anti-cleaved caspase-3 (1:500, Cell Signaling Technologies), rabbit polyclonal anti-Calretinin (1:1000, Abcam #ab702, Cambridge, MA), mouse anti-Reelin (1:1000, Millipore #MAB5366, Burlington, MA), rabbit anti-CCK (1:1000, Frontier Institute Co. Ltd #Af350, Hakaido, Japan), rabbit anti-VIP (1:1000, ImmunoStar #20077, Hudson, Wisconsin). Fluorescent Alexa 488-conjugated antibodies (Alexa Fluor-488-conjugated goat anti-mouse IgG, Alexa Fluor-488-conjugated goat anti-rabbit IgG (1:1000, Life Technologies, Carlsbad, CA) were used as secondary antibodies.

#### Immunostaining

P21 mice were perfused transcardially with 1X phosphate buffer saline (PBS) and fixed with 4% paraformaldehyde. P0 and P5 mice were decapitated and brains were dropped fixed in 4% paraformaldehyde. The brains were postfixed in the same fixative for 1 hour at room temperature for VIP immunostaining and overnight at 4 °C for the all the other immunostaining assays. Postfixed brains were then placed in 30% sucrose solution for dehydration at 4 °C. Coronal sections (50 μm) were cut on a freezing microtome. Tissue sections were permeabilized and blocked in 1 × PBS + 1% bovine serum albumin + 10% normal goat serum + 0.5% Triton X-100 (Carrier PB) at room temperature for 2 h, followed by incubation in primary antibodies, listed above, diluted with 1 × PBS + 1% bovine serum albumin + 1% normal goat serum + 0.1% Triton X-100 overnight at 4 °C. Tissue sections were then incubated with secondary antibodies, listed above, diluted in Carrier Solution (1:1000) at room temperature for 1–2 h and mounted on gelatin-coated slides with Prolong Gold Antifade Mountant (Life Technologies, Carlsbad, CA).

#### Quantitative analysis

Mouse brains from 2–8 different animals were used for each condition. Sections were collected using systematic-random sampling. The 50-μm slices were collected in three parallel sets, each set consisting of 3–6 sections, with each section separated by at least 150 μm. Fluorescent images were captured using the 10X objective of a Zeiss LSM fluorescent microscope and 10 images of hippocampi from each mouse were used to count.

### Electrophysiology

#### Acute slice preparation

Mice between P6-9 (neonatal) and P17-21 (juvenile) were anesthetized with isoflurane and then decapitated. The brain was dissected in ice-cold saline solution (in mM): 130 NaCl, 24 NaHCO_3_, 10 glucose, 3.5 KCl, 1.25 NaH_2_PO_4_, 1 CaCl_2_ and 5 MgCl_2_, saturated with 95% O_2_ and 5% CO_2_, pH 7.4. Horizontal slices (300 µm) were collected from each blocked hemisphere using a vibratome (VT-1000 S Leica Microsystems, Bannockburn, IL, USA). Slices were kept in the same solution until they were transferred to ACSF for recordings.

The artificial cerebrospinal fluid (ACSF) bath solution consisted of the following (in mM): 130 NaCl, 3.5 KCl, 10 D-glucose, 24 NaHCO_3_, 1.25 NaH_2_PO_4_, 2.5 CaCl_2_, 1.5 MgCl_2_, (saturated with 95% O_2_/5% CO_2_ under all conditions, pH 7.4). For feedforward inhibition experiments, CaCl_2_ was increased to 4.5 mM, to enhance presynaptic release probability and the GABA_B_ receptor antagonist, CGP55845 (1 μM) was included.

Recording electrode internal solutions consisted of the following: (1) 130 CsCl, 8.5 NaCl, 5 HEPES, 4 MgCl_2_, 4 Na_2_ATP, 0.3 NaGTP and 5 QX-314 (Tocris), pH 7.2–7.3 for sIPSC and eIPSC recordings; (2) 130 Cs-gluconate, 0.6 EGTA, 10 Bapta, 10 HEPES, 2 MgCl_2_, 2 Na_2_ATP, 0.3 NaGTP and 6 KCl, pH 7.2–7.3 for sEPSC, eEPSC and GDP recordings; and (3) 137 CsCH_3_SO_4_, 4.5 NaCl, 10 HEPES, 4 MgATP, 0.3 NaGTP, 5 QX-314, pH 7.2–7.3, for both eEPSC and eIPSC in feedforward inhibition experiments. Osmolarity was ~290 mOsm for all internal solutions and Biocytin (0.2%) was included for neuronal reconstruction.

All data were filtered at 3 kHz and acquired at a sampling rate of 10 kHz using pClamp9.2 (Molecular Devices, Sunnyvale, CA, USA).

DL-AP5 (50–100 μM) and DNQX (10 μM) were applied to artificial cerebrospinal fluid to isolate spontaneous inhibitory postsynaptic currents (sIPSCs) and picrotoxin (50 μM) was used to confirm the inhibitory nature of the responses. Picrotoxin was also used to isolate sEPSCs and evoked excitatory postsynaptic currents (eEPSCs). NMDAR-mediated EPSCs were isolated with DNQX, in addition to picrotoxin, and confirmed using the NMDAR antagonist DL-APV.

sIPSCs/sEPSCs were detected and analysed in Clampfit using a template-based detection method from gap-free recordings. For recordings of sEPSCs, AMPAR-mediated eEPSCs, sIPSCs, and evoked IPSCs were performed at a holding potential of −70 mV, NMDAR-mediated EPSCs were recorded at +40 or +50 mV. Synaptic events were evoked by use of a glass micropipette filled with ACSF and placed in the alveus for disynaptic feedback inhibition and in CA1 stratum radiatum for NMDA/AMPA currents and disynaptic feedforward inhibition. During feedback inhibition experiments, a field-recording electrode filled with ACSF, placed in stratum pyramidale (SP), was used to record population spikes. DNQX was used to isolate monosynaptic events and these events were digitally subtracted from baseline feedback and feedforward inhibitory waveforms.

### Cell morphology

Slices containing biocytin-filled cells were fixed in 4% paraformaldehyde at 4 °C, then permeabilized and incubated with Alexa-488-conjugated avidin (Molecular Probes, Eugene, OR, USA). Slices were resectioned (70 μm) on a freezing microtome (Microm, Waltham, MA, USA) and mounted using Mowiol (Calbiochem, San Diego, CA, USA) mounting medium. Recorded cells were imaged with Nikon SD microscope at high magnification (40X objective). Three-dimensional reconstruction was performed for each cell using Neurolucida (MBF Bioscience, Williston VT, USA). The number of intersections at concentric circles (20 μm apart) was counted for each cell with the Scholl analysis module of Neurolucida (MBF Bioscience, Williston VT, USA) and analysed using an ANOVA.

### Synaptic quantification

#### Recombinant virus production

PSD-95 with an enhanced green fluorescent protein (EGFP) gene at the C terminus^57^ was cloned into a plasmid carrying a FLEX switch^58^ as described in Akgul *et al*.^59^. FLEX-rev-PSD-95-EGFP_AAV was produced by the University of North Carolina Gene Therapy Program Vector Core.

#### Stereotaxic injections into hippocampus

Mice (P19-22) were anesthetized with isofluorane and were placed into a stereotaxic apparatus (Stoelting, Wood Dale, IL, USA). The fur on the head was removed, and the skin wiped with 70% EtOH. The skull was exposed via a small incision, and a small hole was opened with a dental drill. The following stereotaxic coordinates were used for hippocampus: −2.0 mm anterior to lambda; 2.0 mm lateral from the midline; 1.5 mm down from the dural surface. A pulled glass pipette was mounted, and 0.4 μl of AAV was injected at a rate of 0.1 μl/min into the hippocampus. The syringe was withdrawn 10 min after the final injection. FLEX_PSD-95-EGFP_AAV titer was 1.3 × 10^12^ genome copies per milliliter (GC/ml).

#### Imaging and quantification

14–15 days post-injection, the mouse brains were perfused/fixed with 4% PFA. Cryoprotected brain tissue was sectioned with 50 µm thickness. Mounted sections were imaged using a 63X objective of a Zeiss LSM 880 with Airyscan at the Microscopy and Imaging Core, NICHD. The EGFP positive postsynaptic sites on either soma or dendrites of the infected interneurons images were quantified by making a 3D image reconstitution from Z-stack images and using Spot Detection tool of *IMARIS* software version 8 (Bitplane, Zurich, Switzerland).

### Behavioural analyses

For behavioural testing, we used mixed gender adult mice (8–16 weeks). All tests were done during the light phase. Anxiety-like behaviour was measured using the open field maze. The open field maze comprised a white plastic box (50 cm × 50 cm) with high walls. Mice were allowed to explore inside the box for 60 min. The outer and inner regions on the floor of the box were designated digitally to assess open field anxiety of the mice. Each session was video recorded and analysed using ANY-maze software (Stoelting, Wood Dale, IL, USA).

Social interaction was tested using a three-chamber apparatus (chamber: 20 cm × 40.5 cm × 22 cm). The apparatus was composed of clear plexiglass with doors in the dividing walls, allowing passage. Four 10-min trials were performed in succession for each mouse. First 10-min trial: the mouse was placed in the centre of the apparatus to habituate. Second 10-min trial: the doors were opened and a wire pencil holder representing the novel object was placed in each outside chamber. Third 10-min trial: a wild-type C57/BL6 mouse was placed in a wire pencil holder introducing a social interactor versus an object. Fourth: a wild-type C57/BL6 mouse was placed in a wire pencil holder on the other side of the chamber representing the novel social interactor. Entries into the chambers, as well as distance and immobility measurements were analysed using ANY-maze (Stoelting, Wood Dale, IL, USA).

A Morris Water Maze, was comprised of a pool 1.5 m in diameter. The pool was filled with water (containing food dye to render the water opaque) a day before testing to allow the temperature of the water to come to room temperature. Mice were habituated to the water for 3 days with swimming sessions that lasted 1 min or until they reach the visible platform. During the 5 consecutive days, each mouse was forced to swim in the pool 4 times in search for an invisible submerged platform. At the start of each test epoch the mouse was placed into a different quartile of the pool. On the probe test, the platform was removed from the pool and each mouse was left to swim for 1 min. Each session was video recorded and analysed using ANY-maze software (Stoelting, Wood Dale, IL, USA) and the time and distance the mice took to reach the platform area were used to analyse the working memory of both WT and KO mice.

Data were kept and analyzed in Excel or IGOR Pro (Wavemetrics) version 6.03. Statistical analyses were performed in IGOR Pro or SPSS Statistics (IBM) version 25. For comparisons of two groups, we used Student’s t test (i.e., Fig. 3) and for multiple comparisons, one-way ANOVA (i.e., Figs 1, 4 and 5) or two-way ANOVA(RM) (i.e., Open field maze or Morris water maze, Fig. 7) followed by posthoc Wilcoxon-Mann-Whitney, Bonferroni tests when appropriate. P < = 0.05 is considered as significant. Data were presented as mean ± SEM.

## Data Availability

The datasets generated during and/or analysed during the current study are available from the corresponding author on reasonable request.
